# Accurate Collaborative Globally-Referenced Digital Mapping with Standard GNSS

**DOI:** 10.3390/s18082452

**Published:** 2018-07-28

**Authors:** Lakshay Narula, J. Michael Wooten, Matthew J. Murrian, Daniel M. LaChapelle, Todd E. Humphreys

**Affiliations:** 1 Department of Electrical and Computer Engineering, The University of Texas at Austin, Austin, TX 78712, USA; 2 Department of Aerospace Engineering and Engineering Mechanics, The University of Texas at Austin, Austin, TX 78712, USA; jmwooten@utexas.edu (J.M.W.); matthew.murrian@utexas.edu (M.J.M.); daniel.m.lachapelle@utexas.edu (D.M.L.); todd.humphreys@mail.utexas.edu (T.E.H.)

**Keywords:** vehicle localization, SLAM, sensor fusion

## Abstract

Exchange of location and sensor data among connected and automated vehicles will demand accurate global referencing of the digital maps currently being developed to aid positioning for automated driving. This paper explores the limit of such maps’ globally-referenced position accuracy when the mapping agents are equipped with low-cost Global Navigation Satellite System (GNSS) receivers performing standard code-phase-based navigation, and presents a globally-referenced electro-optical simultaneous localization and mapping pipeline, called GEOSLAM, designed to achieve this limit. The key accuracy-limiting factor is shown to be the asymptotic average of the error sources that impair standard GNSS positioning. Asymptotic statistics of each GNSS error source are analyzed through both simulation and empirical data to show that sub-50-cm accurate digital mapping is feasible in the horizontal plane after multiple mapping sessions with standard GNSS, but larger biases persist in the vertical direction. GEOSLAM achieves this accuracy by (i) incorporating standard GNSS position estimates in the visual SLAM framework, (ii) merging digital maps from multiple mapping sessions, and (iii) jointly optimizing structure and motion with respect to time-separated GNSS measurements.

## 1. Introduction

Localization is one of the primary operations that connected and automated vehicles must perform, both to navigate from one location to another and to interact with each other and with their surroundings within a mapped environment. Satellite-based navigation sensors have historically been the unrivalled sensor of choice for navigating from source to destination. However, the high-reliability sub-50-cm precision demanded by automated vehicles for lane-keeping and other applications, especially in urban areas, has significantly changed this landscape [1]. In most automated vehicles being developed, the Global Positioning System (GPS)/Global Navigation Satellite System (GNSS) is relegated to a secondary sensor whose role is to loosely constrain (within a few meters) the primary localization sensors, usually camera(s) and/or LiDAR, to a global reference frame when building a digital map. The vehicles then locate themselves to decimeter accuracy within this digital map.

Automated driving does not necessarily demand sub-50-cm agreement between the coordinates of a given point in the digital map and the coordinates of the same point in a well-defined global reference frame. Rather, local self-consistency and accurate localization within the digital map is of greater importance. However, consistency of the digital map with a global coordinate frame is likely to become a pre-requisite for cooperative automated driving. Automated driving can be made more efficient (e.g., by platooning) and safe if basic safety information such as vehicle location, velocity, intent, etc. are shared among neighboring agents through vehicle-to-vehicle (V2V) and/or vehicle-to-everything (V2X) communication [2,3]. In many cases, such information can be perceived independently by each agent via its exteroceptive sensors. But in other situations—for example at a blind corner or during a left turn maneuver—full situational awareness will require external sensors—on other vehicles or on infrastructure—that wirelessly communicate data to the ego-vehicle. If all collaborating vehicles navigate within the same digital map, exchange of information can be performed with sub-50-cm precision [4,5], even if the map itself is only globally accurate to a few meters. However, it is unlikely that automated vehicles from different manufacturers will rely on a common digital map. Consequently, the precision of the exchanged vehicle position is lower-bounded by the disagreement on the coordinates of the same physical location between different maps. Thus, exchange of accurate vehicle pose among vehicles, as well as other associated high-level information such as sensor data in the vehicle’s body frame, will demand consistency among, or translation between, different digital maps.

Standard code-phase-based GNSS position measurements, such as those provided by all mass-market GNSS receivers, may be biased by as much as 3–5 meters on any given mapping session. Maps anchored by these measurements may not exhibit lane-level consistency with each other. One possible solution is to create digital maps with decimeter-accurate carrier-phase differential GNSS (CDGNSS) systems [6]. However, at current prices, such systems can only be installed on a limited fleet of specialized mapping vehicles. Precise point positioning (PPP) techniques offer a low-cost alternative to CDGNSS, but the frequent cycle-slipping experienced in urban areas impedes the convergence of PPP techniques [7].

While mapping with a specialized fleet is feasible for urban areas, it is time-consuming and cumbersome to create and maintain maps of entire continents. Thus, a key enabler for large-scale up-to-date maps will be enlisting the help of the very consumer vehicles that need the map to build and update it. Consumer vehicles will likely be equipped with low-cost consumer-grade sensor suites. Accordingly, this paper explores the accuracy limit of globally-referenced mapping involving collaborating consumer vehicles whose sense of global position is based on standard code-phase-based GNSS receivers. Key parameters in this exploration are the asymptotic averages of the error sources that impair code-phase-based GNSS positioning: receiver thermal noise, satellite clock and orbit errors, ionospheric and tropospheric modeling errors, and multipath. One or more vehicles navigating through a digital map over time make multiple time-separated GNSS measurements of the same location. If these vehicles collaboratively update the map over multiple sessions, then the GNSS errors are averaged across all sessions with appropriate weighting.

Are the GNSS errors at every map location—including deep urban locations—asymptotically zero-mean, or, on the contrary, do location-dependent biases persist in averages of time-separated standard GNSS measurements? Such is the question this paper seeks to address. To this end, it describes and demonstrates a stereo-camera-based digital mapping pipeline called GEOSLAM (globally-referenced electro-optical simultaneous localization and mapping)  that achieves the accuracy limit of digital mapping with standard GNSS. GEOSLAM combines standard GNSS position estimates with a visual SLAM system in a tightly-coupled architecture.

To achieve the accuracy limit, GEOSLAM merges and jointly optimizes maps over multiple sessions with time-separated GNSS position estimates. This paper details the techniques GEOSLAM invokes to smoothly transition between unmapped and previously-mapped regions, consistently fusing current and prior maps without the need for a six degrees-of-freedom (6-DoF) pose from an inertial navigation system (INS). GEOSLAM enables multi-agent collaborative mapping by storing and rendering its map in a global frame of reference, such as the World Geodetic System 1984 or the International Terrestrial Reference Frame. Multi-session operation of GEOSLAM is demonstrated using camera and GNSS data collected in a moderate urban environment, and the accuracy of global localization with the multi-session GEOSLAM map is assessed with respect to CDGNSS-based ground truth.

## 2. Previous Work

Improving the accuracy of maps by averaging GPS/GNSS tracks has been explored previously using a variety of approaches. An early effort, detailed in [8], proposed the precise determination of lane centerlines by clustering and averaging the GNSS tracks of probe vehicles. The accuracy of the estimated centerline was assessed in terms of the spread of GNSS tracks, assuming, without analysis, that the error was zero-mean at every location. Likewise, [9] invoked the zero-mean assumption without examination. More recently, [10] proposed vehicle lane determination via PPP on a rural road under open-sky conditions. The current paper aims to perform localization at a similar accuracy level, but in urban and suburban areas and with the aid of a digital mapping sensor.

Minimizing the difference between GNSS measurements and the assigned map coordinates of locations visited multiple times by probe vehicles has been a common feature of the seminal works on map-based precise localization in urban environments for automated driving [4,5], but no analysis of the accuracy of the resulting map in the global coordinate system was provided.

The effect of multipath on measured pseudoranges was studied extensively for various signal types in [11]. However, this study was done under open-sky conditions with a static survey-grade antenna, hardly representative of a mass-market receiver in an urban environment. The effect of multipath from a single large reflector on a static receiver was studied in [12], where it was shown that the position solution of the receiver was significantly biased when tracking multipath-afflicted signals. However, no details were provided about the tracking strategy and navigation filter. Multipath characterization for a dynamic receiver in an urban area was performed in [13], but the study was carried out for a single run through the test area, and many of the important error sources (e.g., atmospheric modeling errors), were assumed to be negligible. A detailed study on the distribution of code-phase and Doppler offsets of the multipath components from individual satellites in a dynamic urban setting was carried out in [14]. However, the error was characterized as the combined distribution of code phase delays over the entire duration of the run, which marginalizes over the temporally- and physically-local biases. On the contrary, this paper explores the errors in the position domain for repeated sessions through a given realization of an urban corridor.

Other GNSS error sources such as errors in modeling of ionospheric [15] and tropospheric [16] delay have been studied extensively over many decades, and their long-term error characteristics have also been reported in the literature. However, the impact of these errors on the asymptotic statistics of code-phase-based GNSS position estimates has not been previously presented.

To the authors’ best knowledge, despite the apparent simplicity of the problem, no prior work has studied the long-term statistics of GNSS errors in an urban environment representative of the conditions to be encountered by consumer vehicles creating digital maps anchored by code-phase-based GNSS positioning. One of this paper’s primary contributions is to address this gap in the literature.

Sensor fusion of visible-light cameras and GNSS has been extensively studied  [17,18,19,20,21,22,23,24,25,26,27,28]. Some of these works [26,27,28] have proposed visual odometry as a replacement for, or an augmentation of, the traditional GNSS/INS architecture. Visual data from cameras are exploited to perform dead reckoning in a visual odometry pipeline, wherein an important distinction from the current paper is that the 3D map points do not persist after a window of time has elapsed—that is, no map of feature points is maintained. Clearly, such an approach does not allow improvement of the 3D map point positions over multiple mapping sessions.

In [25], the relative change in position between two image frames is first estimated based on time-differenced GNSS carrier phase measurements. The metric-accurate GNSS-derived change in position is exploited to initialize the otherwise unobservable scale in a monocular visual SLAM system. However, GNSS measurements are not incorporated any further once the absolute scale has been initialized. Unlike the current paper, the visual SLAM map is rendered in the arbitrary SLAM coordinate frame since only the relative change in position, and not the absolute position, was estimated based on GNSS measurements.

The vision-GNSS fusion in the current paper is closely aligned with the bi-objective bundle adjustment (BA) optimization techniques previously reported in [17,18,20,22,23,24]. In [17,18,20,22], the traditional visual SLAM reprojection cost function is jointly minimized along with a GNSS position error term. The methods proposed in [23,24] are also similar, but guarantee that the visual reprojection cost after incorporation of the GNSS term is not significantly greater than the visual-only case. However, none of these works showed significant empirical evidence of their efficacy on real-world vehicle data sets. Furthermore, collaborative mapping or multi-session improvement of the map was not discussed.

Collaborative multi-agent mapping, without GNSS aiding, has also been extensively discussed in the literature [29,30,31,32,33,34,35]. Some of these proposed solutions require significant overlap in the field-of-view of the agents, or require that the relative pose transformation between the agents be known a priori [30,32]. Other solutions, such as in [31,33,34,35], enable collaboration by performing data association between non-concurrent mapping sessions where the relative pose transformation between the agents is unknown. The multi-session strategy employed in this paper is similar, but with an important distinction: none of the previous works on collaborative mapping have incorporated GNSS measurements in the map-building process. Without global referencing, the problem of data association between non-concurrent sessions becomes intractable. With no estimate of the pose for the mapping platform in relation to the existing map, data association must be attempted against the entire map. It is easily observed that such data association will become infeasible when scaled to city- or country-wide maps. The current paper proposes rendering and storage of digital maps in a global coordinate frame, such that a new mapping session can readily estimate its approximate pose in relation to the prior map, and perform data association on a small segment of the prior map that is expected to be in view of the vision system.

The work presented in [36] is perhaps the most closely related to the current paper. In [36], a particular stretch of roadway is mapped 25 times with a low-cost sensor setup. However, [36] assumes, without detail, the availability of a lane-level accurate low-cost positioning module that provides the full 6-DoF pose for the mapping platform. This greatly simplifies the ensuing data association and mapping pipeline. No mention is made of the general setting of the roadway being mapped (open-sky highway, urban canyon, etc.), and while the accuracy of the mapped traffic signs is adequately reported, localization within the map is not discussed, presumably since lane-level accurate positioning is already available. Meanwhile, the current paper only assumes the availability of a meter-level accurate code-phase-based GNSS receiver that provides 3D position estimates. Global localization accuracy of a vehicle operating within the multi-session map is presented as the key performance indicator.

## 3. GNSS Error Analysis

### 3.1. Low-Cost GNSS in Urban Areas

Low-cost multi-GNSS receiver manufacturers have recently announced the development and release of low-cost multi-frequency multi-GNSS receivers [37]. Accordingly, the analysis in this section considers a vehicular platform equipped with a multi-frequency multi-GNSS receiver capable of tracking both code and carrier phase of GNSS signals.

Development of an extensive dense reference network in support of CDGNSS consumer vehicular positioning in urban areas, as suggested in [38], could be an expensive affair. PPP is a low-cost alternative to CDGNSS that requires only a sparse network of reference stations across the globe, but is not considered a viable option for urban GNSS positioning in this paper because the constant cycle slips and outages experienced in urban areas [6] make it difficult for PPP’s float carrier phase ambiguity estimates to converge [7], in which case PPP degrades to code-phase positioning accuracy.

While convergence of PPP carrier-phase ambiguities may be infeasible in urban areas, a partial PPP solution that exploits precise satellite orbits and clocks, as well as ionospheric and tropospheric corrections, can certainly improve the accuracy of code-phase-based GNSS position estimates. Since connected and automated vehicles will perforce enjoy network connectivity, this paper assumes the availability of such GNSS corrections. Thus, the kind of GNSS errors assessed in this section lie between those corresponding to the two extremes of standard standalone code-phase positioning and PPP. This type of GNSS positioning, hereafter referred to as enhanced code-phase positioning, exploits both code and carrier phase or frequency tracking, but, as opposed to PPP, does not attempt to estimate a quasi-constant float carrier phase ambiguity, making it suitable for urban applications.

### 3.2. Pseudorange Measurement

The pseudorange measurement at receiver R from satellite $S_{i}$ is modeled as

(1) $$\begin{array}{cc} \rho_{i}\left(t_{R}\right) & =h_{i}\left[x\left(t_{R}\right),I_{\rho_{i}}\left(t_{R}\right),T_{i}\left(t_{R}\right),t_{R}\right]+w_{\rho_{i}}\left(t_{R}\right)\\ & =\Delta r_{i}+c\left[\delta t_{R}\left(t_{R}\right)-\delta t_{S_{i}}\left(t-\delta t_{{\mathrm{TOF}}_{i}}\right)\right]+I_{\rho_{i}}\left(t_{R}\right)+T_{i}\left(t_{R}\right)+w_{\rho_{i}}\left(t_{R}\right), \end{array}$$

where

$$x\left(t_{R}\right)≜\left[\begin{array}{c} r_{R}\left(t_{R}\right)\\ \delta t_{R}\left(t_{R}\right) \end{array}\right]$$

is the state of the receiver, comprising the receiver position, $r_{R}\left(t_{R}\right)$, at the time of the signal receipt event, $t_{R}$, and the receiver clock bias, $\delta t_{R}\left(t_{R}\right)=t_{R}-t$, with respect to true time *t*. The nonlinear measurement model is denoted by $h_{i}$; $\rho_{i}$ denotes the measured pseudorange to $S_{i}$; *c* denotes the speed of light in vacuum; $\delta t_{S_{i}}\left(t\right)=t_{S_{i}}-t$ denotes the satellite clock bias with respect to *t*; $\delta t_{{\mathrm{TOF}}_{i}}$ denotes the time-of-flight of the signal from $S_{i}$, as an increment in true time; $I_{\rho_{i}}$ and $T_{i}$ denote the ionospheric and tropospheric delay experienced by the signal from $S_{i}$, respectively; $w_{\rho_{i}}∼\left(\mu_{w_{i}},\sigma_{w_{i}}^{2}\right)$ denotes the sum of measurement thermal noise, multipath interference, non-line-of-sight (NLOS) delay, and other unmodeled errors; and $\Delta r_{i}$ denotes the true range between R and $S_{i}$, given as

$$\Delta r_{i}=∥r_{R}\left(t_{R}\right)-r_{S_{i}}\left(t_{R}-\delta t_{R}\left(t_{R}\right)-\delta t_{{\mathrm{TOF}}_{i}}\right)∥,$$

where $r_{S_{i}}$ is the satellite position at the signal transmit event. Note from (1) that the receiver clock bias component of the state contributes identically to all pseudorange measurements.

Taking $n_{z}$ pseudorange measurements ${\left\{\rho_{i}\right\}}_{i=1}^{n_{z}}$ and predictions ${\bar{I}}_{\rho_{i}}$ and ${\bar{T}}_{i}$ for each measurement, R estimates its state by solving a nonlinear least squares problem based on (1). First, it linearizes the measurement model in (1) about an initial guess of its state $\bar{x}\left(t_{R}\right)={\left[{\bar{r}}_{R}^{T}\left(t_{R}\right)\;{\bar{\delta t}}_{R}\left(t_{R}\right)\right]}^{T}$ and the modeled atmospheric delays:

$$\begin{array}{cc} \rho_{i} & \approx h_{i}\left(\bar{x},{\bar{I}}_{\rho_{i}},{\bar{T}}_{i},t_{R}\right)+\underset{H_{i}}{\underset{︸}{{\left[\frac{\partial h_{i}}{\partial x}\right]}_{x=\bar{x}}^{T}}}\left(x-\bar{x}\right)+{\tilde{I}}_{\rho_{i}}+{\tilde{T}}_{i}+w_{\rho_{i}}, \end{array}$$

with ${\tilde{I}}_{\rho_{i}}=I_{\rho_{i}}-{\bar{I}}_{\rho_{i}}$ and ${\tilde{T}}_{i}=T_{i}-{\bar{T}}_{i}$. Representing all $n_{z}$ measurements in matrix form yields

$$\begin{array}{cc} \left[\begin{array}{c} \rho_{1}\\ \vdots \\ \rho_{n_{z}} \end{array}\right] & =\left[\begin{array}{c} h_{1}\left(\bar{x},{\bar{I}}_{\rho_{1}},{\bar{T}}_{1},t_{R}\right)\\ \vdots \\ h_{n_{z}}\left(\bar{x},{\bar{I}}_{\rho_{n_{z}}},{\bar{T}}_{n_{z}},t_{R}\right) \end{array}\right]+\left[\begin{array}{c} H_{1}\\ \vdots \\ H_{n_{z}} \end{array}\right]\left(x-\bar{x}\right)+\left[\begin{array}{c} {\tilde{I}}_{\rho_{1}}\\ \vdots \\ {\tilde{I}}_{\rho_{n_{z}}} \end{array}\right]+\left[\begin{array}{c} {\tilde{T}}_{1}\\ \vdots \\ {\tilde{T}}_{n_{z}} \end{array}\right]+\left[\begin{array}{c} w_{1}\\ \vdots \\ w_{n_{z}} \end{array}\right] \end{array}$$

or

(2) $$\begin{array}{cc} \rho & =h\left(\bar{x},\bar{I},\bar{T},t_{R}\right)+H\left(x-\bar{x}\right)+\tilde{I}+\tilde{T}+w. \end{array}$$

Rearranging measured and modeled quantities on the left-hand side to get the standard form for a linearized measurement model yields

(3) $$\begin{array}{c} z≜\rho -h\left(\bar{x},\bar{I},\bar{T},t_{R}\right)+H\bar{x}=\Rightarrow z=Hx+\tilde{I}+\tilde{T}+w. \end{array}$$

The *i*th row of the measurement sensitivity matrix *H* is

$$\begin{array}{cc} H_{i} & \approx \left[\begin{array}{cc} \frac{{\bar{r}}_{R}^{T}\left(t_{R}\right)-{\bar{r}}_{S_{i}}^{T}\left(t_{R}-{\bar{\delta t}}_{R}-{\bar{\delta t}}_{{\mathrm{TOF}}_{i}}\right)}{∥{\bar{r}}_{R}\left(t_{R}\right)-{\bar{r}}_{S_{i}}\left(t_{R}-{\bar{\delta t}}_{R}-{\bar{\delta t}}_{{\mathrm{TOF}}_{i}}\right)∥}, & 1 \end{array}\right]. \end{array}$$

By solving (3) for x, updating $\bar{x}$, and iterating until convergence, R obtains its state estimate $\hat{x}\left(t_{R}\right)$:

$$\hat{x}\left(t_{R}\right)≜\left[\begin{array}{c} {\hat{r}}_{R}\left(t_{R}\right)\\ {\hat{\delta t}}_{R}\left(t_{R}\right) \end{array}\right].$$

For dynamic applications such as vehicle tracking, the state $x(t_{R})$ is typically augmented to include the time derivatives of $r_{R}\left(t_{R}\right)$ and $t_{R}\left(t_{R}\right)$, and the measurement model typically assumes direct measurement of apparent Doppler frequency.

### 3.3. Error Sources

The major sources of error in the estimates ${\hat{r}}_{R}$ and ${\hat{\delta t}}_{R}$ are as follows:

#### 3.3.1. Thermal Noise

Measurement thermal noise at the receiver is one of the components of $w_{\rho_{i}}$ in (1). The effect of thermal noise can be accurately modeled as a white Gaussian random variable with zero mean and standard deviation $\sigma_{T}$. For the pseudorange measurement, $\sigma_{T}$ is typically between 10–30 cm, depending on the signal carrier-to-noise ratio, signal bandwidth, and receiver tracking bandwidth [39]. Estimation of the receiver state from multiple appropriately-weighted measurements with independent thermal-noise errors, and processing such measurements over time through a filter based on the modeled dynamics of the receiver, renders negligible the position-domain effects of uncorrelated zero-mean thermal noise. As a result, thermal noise is not a major contributor to the asymptotic accuracy of a digital map.

#### 3.3.2. Satellite Orbit and Clock Errors

Satellite orbit and clock errors manifest in the modeled satellite position ${\bar{r}}_{S_{i}}$ and the modeled satellite clock bias ${\bar{\delta t}}_{S_{i}}$. The International GNSS Service (IGS) provides orbit and clock models for GNSS satellites. The predicted *ultra rapid* orbits and satellite clocks have an accuracy of ∼5 cm and ∼3 ns, respectively [40]. These may add up to ∼1 m of combined pseudorange model error for a given satellite. The 17-h retroactively-available *rapid* orbits and satellite clock models are accurate to ∼2.5 cm and ∼75 ps RMS errors, respectively [40], adding up to less than 5 cm of RMS error in the modeled pseudorange for a given signal. Since the orbit and clock parameters are fit to measurements made at IGS analysis centers, the errors in the estimated parameters must be asymptotically zero-mean by design of the estimator. For post-processing applications such as mapping, it is reasonable to assume the availability of *rapid* orbit and satellite clock products, and thus the asymptotic average position errors due to errors in modeled satellite position and clock bias can be reduced to a sub-5-cm level.

#### 3.3.3. Ionospheric Modeling Errors

The code-modulated GNSS signal propagates slower through the ionosphere as compared to vacuum due to the slightly-greater-than-unity *group* index of refraction for this atmospheric layer. The excess group delay is given as

$$\Delta \tau_{g}=\frac{40.3\cdot TEC}{cf^{2}},$$

where TEC is the total electron content in electrons/m${}^{2}$ and *f* is the frequency of the propagating signal. At the GPS L1 frequency centered at 1575.42 MHz, the excess ionospheric group delay is roughly 16.24 cm per TECU (1 TECU ≜ 10${}^{16}$ electrons/m${}^{2}$). If not modeled, the ionospheric delay can lead to ranging errors greater than 15 m.

The ionospheric delay can be estimated via an ionosphere model or, in case of a multi-frequency receiver, eliminated via a combination of multiple-frequency pseudorange measurements. The latter technique does not require any external aiding, but the formation of the ionosphere-free combination exacerbates pseudorange noise, including any biases due to tracking of multipath signals. Compensating for ionospheric delay with the aid of an ionosphere model is applicable to both single- and multi-frequency receivers. It relies on accurate delay modeling based on ionospheric measurements at permanent GNSS reference stations, such as those that form the IGS network. While both methods have their merits, the analysis in this section considers corrections from an ionospheric model, and thus will not be relevant to applications where the ionosphere-free combination is applied. Note that those applications would likely experience worse multipath errors than the ones presented later, requiring a separate multipath analysis along the lines of Section 3.3.5.

Ionospheric model accuracy was studied comprehensively in [15]. The method in [15] generates unambiguous carrier-phase measurements from a global distribution of permanent receivers to compute the true slant total electronic content (STEC) for each satellite, and compares the model prediction for a number of models with the ground truth. In [41], the same authors compared PPP convergence times when applying different ionospheric correction models. This section extends the analysis in [15,41] to examine whether there exist long-term position-domain biases in enhanced code-phase positioning.

The post-fit residuals for multiple regional and global ionospheric models, computed as described in [15], were graciously made available by the same authors for the year 2014. These residuals were computed for GPS signals as observed at about 150 reference stations around the globe at 5 min intervals.

To observe the position-domain effect of the ionospheric modeling errors in isolation, this section neglects all other error sources, reducing the linearized measurement model in (3) to

$$z=Hx+\tilde{I}.$$

Historical GPS satellite almanacs can be combined with the timestamps from the residuals data to obtain the measurement sensitivity matrix *H* at each epoch for each station. With an elevation-dependent measurement covariance matrix *R*, the error in the weighted least-squares solution due to errors in ionospheric modeling is

$$\hat{x}-x={\left(H^{T}R^{-1}H\right)}^{-1}H^{T}R^{-1}\tilde{I}.$$

**Figure 1:**
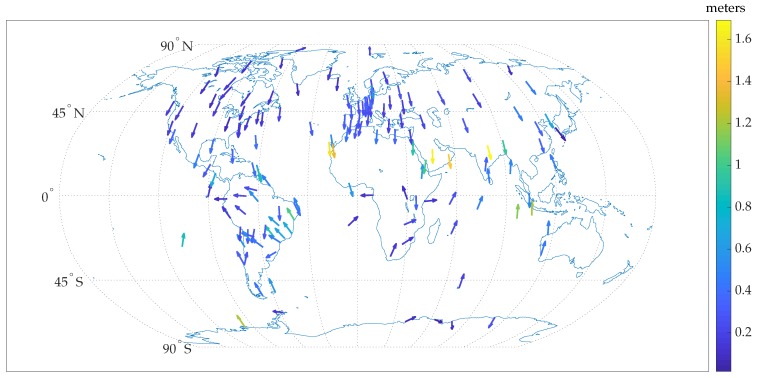
Direction and magnitude (the latter represented by color, in meters) of the long-term average horizontal position error due to errors in the delay estimates provided by the IGS GIM. Note that the meridians are curved outwards due to projection of the spherical map, and that arrows parallel to the curved meridians point directly south or north.

Figure 1 summarizes the results for ionospheric corrections obtained from the IGS global ionospheric map (GIM). Each of the arrows in Figure 1 points in the direction of the position bias in the east-north plane, as estimated over 12 months of data from 2014 (more than 800,000 samples per station). The magnitude of the horizontal position bias is depicted by the color of the arrow according to the scale shown on the right. Interestingly, there is a clear trend of southward bias in the position error for most stations in the northern hemisphere, and a mild trend of northward bias in the position error for stations in the southern hemisphere. A numerical summary of the IGS GIM position bias is presented in Table 1, along with a similar analysis for the Wide Area Augmentation System (WAAS) ionospheric corrections available for the contiguous United States (CONUS) region. As reported in [15], the WAAS model was found to exhibit a significantly smaller RMS error in ionosphere TEC estimates when compared to the IGS GIM; however the long-term position bias due to WAAS corrections is similar to or worse than those for the IGS model.

Another global ionospheric model, the Fast PPP model [41], was also studied as above. Fast PPP natively models the ionosphere as a two-layered shell, but is also made available in the standard one-layer IONEX (ionosphere-map exchange) format [15] for dissemination. The results presented in Table 1 represent the IONEX version of Fast PPP. In comparison with the IGS corrections, it is clear that the Fast PPP IONEX GIM corrections result in substantially unbiased long-term position errors at the global test locations. However, it must be conceded that the results in Table 1 are best-case results, as they are based on data from the same permanent reference stations used to constrain the model.

**Figure 2:**
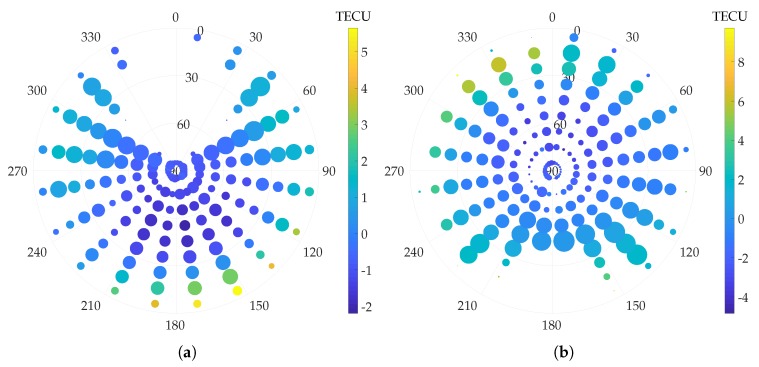
Azimuth and elevation dependence of post-fit IGS global ionospheric map (GIM) residuals. (**a**) A representative station from the northern hemisphere. (**b**) A representative station from the southern hemisphere. The average residual error (in TECU) is denoted by the color of the disc. The size of the disc indicates the number of samples of post-fit residuals available in each bin.

To understand the reason behind the systematic biases with IGS corrections, note that any ionospheric modeling bias that identically affects all satellites does not have any impact on the accuracy of the GNSS position solution, as this common error is absorbed in ${\hat{\delta t}}_{R}$. Rather, position-domain biases arise from the azimuthal- and elevation-dependence of ionosphere model errors. From analysis of the spatial distribution of post-fit residuals, it was found that appreciable azimuthal and elevation residual gradients persist in the IGS ionospheric corrections. These gradients are represented graphically in Figure 2 for one representative station from the northern hemisphere (station code: EUSK, latitude: 50${}^{\circ }$40${}^{\prime }$26.87${}^{\prime \prime }$, longitude: 6${}^{\circ }$45${}^{\prime }$48.72${}^{\prime \prime }$) and one representative station from the southern hemisphere (station code: VACS, latitude: -20${}^{\circ }$17${}^{\prime }$48.47${}^{\prime \prime }$, longitude: 57${}^{\circ }$29${}^{\prime }$13.79${}^{\prime \prime }$). The post-fit residuals are binned in azimuth and elevation and the average value in each bin is denoted by the color of the representing disc. The size of the disc denotes the number of samples of post-fit residuals available in each bin. Due to the inclination angle of the GPS satellite orbits, the angular distribution of satellites at any given latitude is non-uniform.

From Figure 2, it is clear that the elevation gradients in the ionospheric residuals are pronounced. A subtle azimuthal gradient also exists, mainly along the north-south direction. Such spatial non-uniformity, coupled with the non-uniform satellite angular distribution, may be the reason for the observed persistent position biases. While the elevation gradients are consistent for stations at all locations, the azimuthal gradients appear to invert along the north-south direction between the northern and southern hemisphere. This is likely the reason for the opposite direction of the average horizontal position bias in the northern and southern hemispheres.

Such persistent position-domain biases due to inaccurate ionospheric modeling have not been previously reported in the literature, and are a rather remarkable result. While some single-frequency PPP (SF-PPP) techniques eliminate the ionospheric delays based on the GRAPHIC combination [42], many other techniques that rely solely on ionospheric corrections from GIMs have been shown to achieve 30 cm 95% accuracy in the east-north plane after convergence, with sub-10-cm bias [43], seemingly contradicting the results here. The key difference is that the SF-PPP methods involve estimation of a float carrier ambiguity term for each satellite arc. A portion of the systematic biases in the GIM estimates is likely absorbed in these states of the estimator, thereby attenuating the position biases in the east-north plane. For instance, the SF-PPP technique in [43] is based on the phase-adjusted pseudorange algorithm [44], wherein the ambiguity term for each satellite, physically an unknown constant, is in fact iteratively estimated with small but non-zero process noise. In such an estimator, the ambiguity term can absorb slowly time-varying systematic biases. In other SF-PPP techniques, the ionospheric correction term is explicitly included as a state to be estimated, and the estimates from GIM are applied as pseudo-observations [45,46]. Once again, decimeter-level biases in the GIM estimates of the ionospheric delay may not necessarily appear in the final reported position accuracy of the SF-PPP method. Of course, such absorption of biases in augmented states is not undesirable. However, for the case of urban vehicular positioning, convergence of SF-PPP is a concern due to carrier phase cycle slipping, as discussed earlier. In an enhanced code-phase-based receiver, the high variance of the code noise leads to poor observability of the decimeter-level horizontal position bias due to ionospheric modeling errors. Thus, ionospheric biases are not often estimated in a code-phase-based GNSS estimator.

Another factor of note is that 2014 was a maximum in the 11-year solar activity cycle, and thus the IGS GIM accuracy may have been worse than usual over this period of time.

In conclusion, persistent decimeter-level biases in the east-north plane and meter-level biases in the vertical direction can arise when ionospheric delay corrections are sourced from the IGS GIM, or similar, even under ideal open-sky conditions. More advanced models of the ionosphere with more accurate slant TEC measurements may achieve better results. Elimination of the ionospheric delay based on the ionosphere-free combination is another option, but tends to worsen multipath-induced position errors. If corrections from some ionosphere model lead to unbiased position errors, then for globally-referencing digital maps by averaging GNSS measurements over many sessions it is advisable to avoid the combination of multi-frequency signals.

#### 3.3.4. Tropospheric Modeling Errors

In the troposphere, or more generally the neutral atmosphere, the index of refraction departs from unity much less than in ionosphere at GNSS frequencies, causing a delay of ∼2.4 m at zenith. The index of refraction in the troposphere is non-dispersive, and thus cannot be estimated using multiple-frequency signals. The tropospheric delay is obtained from models of the climatological parameters (temperature, pressure, and water vapor pressure) along the propagation path.

State-of-the-art tropospheric models [16] fit a small number of location- and day-of-year-dependent coefficients to climatological data from numerical weather models (NWMs) to model the zenith tropospheric delay. The zenith delay is mapped to any elevation angle using mapping functions [47]. Similar to the ionospheric models, the tropospheric mapping functions may introduce a differential azimuth- and elevation-dependent error. For empirically-derived mapping functions such as VMF1 [47] and GMF [48], the mean error at lowest elevation of 5${}^{\circ }$ has been shown to be under 50 mm (this value is typically reported as 10 mm station height error, which is approximately one-fifth of the delay error at lowest elevation [47]). As a result, this paper assumes that time-averaged tropospheric model errors would introduce sub-5-cm errors in the position domain, and would thus not impede asymptotically accurate collaborative mapping in both horizontal and vertical components at the several-decimeters level.

#### 3.3.5. Multipath Error

In ideal circumstances, each signal received from an overhead satellite arrives only along the least-time path. In practice, however, this so-called line-of-sight (LOS) component is accompanied by other components due to signal diffraction and single- or multiple-signal reflections off surrounding surfaces and obstacles (e.g., the glass facade of a nearby building, poles, trees, etc.). The complex baseband representation of the *N* signal components received from a particular satellite at a particular frequency and code is

$$r\left(t\right)=\sum_{i=0}^{N-1}A_{i}\left(t\right)C\left[t-\tau_{i}\left(t\right)\right]\exp\left[j\theta_{i}\left(t\right)\right],$$

where $A_{i}$ is the amplitude of the *i*th component, C(t) is the GNSS code modulation, $\tau_{i}\left(t\right)$ is the delay of the *i*th signal component relative to an unobstructed LOS signal, and $\theta_{i}\left(t\right)$ is the beat carrier phase of the *i*th component. The combination of multiple components distorts the received signal and causes errors in the pseudorange and phase measurements.

Unlike the study of ionospheric modeling errors, for application in urban mapping, multipath errors cannot be characterized with data from survey stations with a clear view of the sky. This section considers a simulation approach for scalable analysis of multipath tracking errors in an urban environment. The objective of this study was to inspect the presence of persistent biases caused by multipath due to the surrounding structure in the navigation solution averaged over multiple sessions

##### Scenario Setup

**Figure 3:**
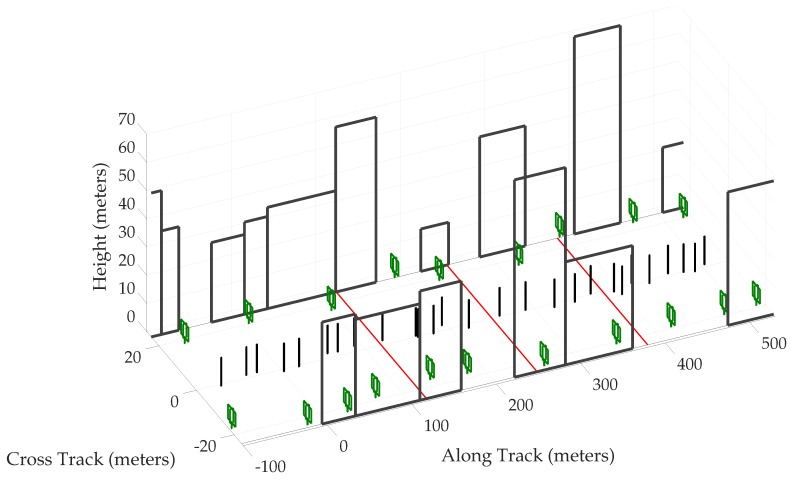
Initial segment of the simulated urban corridor. Red lines across the road denote the positions where the vehicle is momentarily stopped.

The present simulation study was based on the open-access Land Mobile Satellite Channel Model (LMSCM) [49], itself based on extensive experimentation with a wideband airborne transmitter at GNSS frequencies in urban and suburban environments. First, an urban corridor was simulated stochastically following the procedure described in [50]. The corridor was composed of buildings, trees, and poles. Some of the important parameters for the generation of the scene are summarized in Table 2, and a part of the scene realization is shown in Figure 3. Multi-GNSS satellite trajectories were generated at randomly-selected times based on GPS and Galileo satellite almanac data. An average of 25 satellites were available above an elevation mask of 5${}^{\circ }$, consistent with modern multi-GNSS receivers. The satellites were assumed to be stationary over the simulation period of 60 s. Navigation solution errors were computed over 1000 60-s sessions.

**Figure 4:**
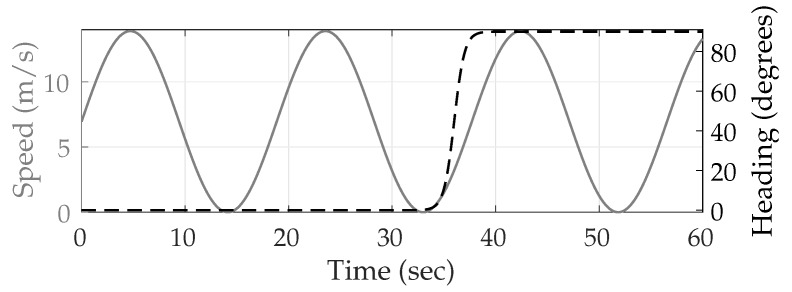
Vehicle speed (solid line) and heading (dashed line) simulating stop-and-go motion with a $90^{\circ }$ right turn.

The vehicle trajectory was kept consistent across all 1000 driving sessions to avoid decorrelation of multipath error due to variable receiver motion. The trajectory was parametrized by its speed and heading, as described in [50]. The vehicle started at the zero coordinate on the along-track axis, and traveled in the positive direction, which was assumed to be aligned with the local north. The simulated trajectory was 60 s long and simulated a vehicle in stop-and-go traffic executing one 90${}^{\circ }$ right turn, as shown in Figure 4. The vehicle traveled roughly 430 m and faced eastwards at the end of the trajectory. The three low-speed intervals, marked with red line segments in Figure 3, are expected to present severe multipath effects since multipath errors decorrelate slowly, and thus tend to reinforce one another within the navigation filter, when the vehicle moves slowly.

##### Multipath Simulation

The LMSCM generates power, delay, and carrier phase for *N* LOS and echo signals. The interaction of the LOS with the simulated obstacles is governed by deterministic models for attenuation, diffraction, and delay. The LOS components of the combined signal, denoted $r_{\mathrm{LOS}}\left(t\right)$, may be composed of multiple components due to signal diffraction. These components are modeled as

$$r_{\mathrm{LOS}}\left(t\right)=\sum_{i=0}^{N_{\mathrm{LOS}}-1}A_{i}\left(t\right)C\left[t-\tau_{i}\left(t\right)\right]\exp\left[j\theta_{i}\left(t\right)\right].$$

In the special case of an unobstructed LOS signal, $N_{\mathrm{LOS}}=1$, $A_{0}\left(t\right)=1$, $\tau_{0}\left(t\right)=0$, and

$$\theta_{0}\left(t\right)=\frac{∥r_{R}\left(t\right)-r_{S}\left(t\right)∥\cdot 2\pi }{\lambda }+\gamma_{0},$$

where λ denotes the wavelength and $\gamma_{0}$ is a constant due to phase initialization in the satellite and receiver [51].

The LMSCM generates the $N-N_{\mathrm{LOS}}$ NLOS echoes stochastically based on satellite azimuth and elevation, receiver dynamics, and general characteristics of the scene (e.g., an *urban car* scenario). This stochastic procedure might not be representative of multipath over multiple sessions through the same urban corridor, where certain echoes might persist over different sessions. To address this limitation, the LMSCM was augmented by the present authors to generate one- and two-bounce deterministic reflective NLOS echoes off the simulated buildings, and a one-bounce NLOS echo off the ground surface. These three additional reflective NLOS echoes, denoted $r_{\mathrm{DET}}\left(t\right)$, were added to r(t) and are modeled as

$$r_{\mathrm{DET}}\left(t\right)=\sum_{i=N}^{N+2}b_{i}\left(t\right)A_{i}\left(t\right)C\left[t-\tau_{i}\left(t\right)\right]\exp\left[j\left(\theta_{i}\left(t\right)+\theta_{i}^{\prime }\left(t\right)\right)\right],$$

where $b_{i}\left(t\right)\in \left\{0,1\right\}$ denotes whether the surrounding geometry supports the reflective echo. Since these reflections are expected to be the stronger than other diffracted and multiple-bounce NLOS echoes, the amplitudes $A_{i}\left(t\right),i\in \left\{N,N+2\right\}$ for reflective echoes were drawn from the distribution of the strongest echo generated stochastically by the LMSCM at each epoch. By experiment, this distribution was found to be log-normal with $20\log_{10}\left(A_{i}\right)∼N\left(-22,5\right),i\in \left\{N,N+2\right\}$. The delays for the reflective echoes are given as

$$\tau_{i}\left(t\right)=\frac{∥r_{R}^{\prime }\left(t\right)-r_{S}\left(t\right)∥-∥r_{R}\left(t\right)-r_{S}\left(t\right)∥}{c},\;i\in \left\{N,N+2\right\},$$

where $r_{R}^{\prime }\left(t\right)$ is the position of the imaginary *image* antenna [52] about the reflecting plane (building or ground). Similarly, the carrier-phase of the reflective echoes is computed geometrically as

$$\theta_{i}\left(t\right)=\frac{∥r_{R}^{\prime }\left(t\right)-r_{S}\left(t\right)∥\cdot 2\pi }{\lambda }+\gamma_{0},\;i\in \left\{N,N+2\right\}.$$

A random carrier-phase offset $\theta_{i}^{\prime }\left(t\right)\in \left[0,2\pi \right)$ was added at the reflection point every time a new reflective echo was spawned to simulate the material-specific phase offset introduced by the reflection process.

##### Receiver

A receiver simulator was developed to account for the mediating effects that a receiver’s tracking loops and navigation filter have on multipath-induced position errors in a receiver’s reported position solution. The simulated receiver tracks the combination of all $N_{\mathrm{LOS}}$ line-of-sight signals and $N+2-N_{\mathrm{LOS}}$ multipath echoes for a given signal. If R(τ) denotes the correlation function of the GNSS signal’s spreading code, then the multipath delay error in the tracked code phase, relative to unobstructed LOS, is given as the solution to [52]

$$\begin{array}{cc} 0=S_{\mathrm{coh}}\left(\tau \right) & =\sum_{i=0}^{N+2}A_{i}\cos\left(\theta_{i}-\theta_{c}\right)\\ & \;\times \left[R\left(\tau -\tau_{i}+\frac{d}{2}\right)-R\left(\tau -\tau_{i}-\frac{d}{2}\right)\right], \end{array}$$

where $\theta_{c}$ is the tracked carrier-phase of the combined signal:

$$\theta_{c}=\mathrm{atan}2\left(\sum_{i=0}^{N+2}A_{i}R\left(\tau_{c}-\tau_{i}\right)\sin\left(\theta_{i}\right),\sum_{i=0}^{N+2}A_{i}R\left(\tau_{c}-\tau_{i}\right)\cos\left(\theta_{i}\right)\right).$$

The paramter *d* is the early-to-late correlator spacing in the receiver. It is well-known that a wide-bandwidth receiver with narrow correlator spacing mitigates the effect of multipath [52]. To this end, the receiver considered in this simulation implements d=0.1. It must be mentioned that R(τ) was implemented as the correlation function for GPS L1 C/A identically for all the simulated signals. Modernized GNSS signals have better multipath mitigation characteristics [11], but this behavior was not included in the simulation.

Another important observation is that when the LOS signal is strong as compared to the echo signals, the time derivative of the tracked carrier-phase is equal to the Doppler frequency of the LOS signal, which changes smoothly in accordance with the motion between the satellite and the receiver. However, when the LOS signal is comparable to or weaker than other rapidly-decorrelating echoes, the combined carrier-phase is uniformly random. In a GNSS receiver, the phase lock loop’s phase-lock indicator indicates whether a sufficiently strong LOS signal is available, enabling carrier lock [6]. The simulator’s phase-lock indicator is asserted only if (1) the tracked Doppler frequency does not deviate significantly from a second-order polynomial, and (2) the strongest received echo is attenuated by more than 25 dB with respect to an unattenuated signal.

##### Navigation Filter

At each epoch, $n_{z}$ multipath-free, ionosphere-free, and troposphere-free simulated pseudorange measurements were combined with corresponding simulated multipath tracking delay errors and fed to a navigation filter that estimates the receiver state. The navigation filter implemented in this paper is an extended Kalman filter (EKF) with a nearly constant velocity motion model following [53]. The standard details of the EKF are omitted for brevity.

The effect of multipath tracking on the navigation solution is strongly dependent on the receiver’s multipath rejection scheme. Two schemes are explored here. The first is a hypothetical ideal multipath rejection scheme that excludes all signals for which the LOS signal has a smaller-than-10-dB advantage over its multipath echoes. The second scheme implements a normalized innovation squared (NIS) test to reject multipath signals based on measurement innovations [53]. At the (k+1)th measurement update step, the difference between the predicted and observed measurement vector, called the innovation and denoted ν(k+1), is squared and normalized by its covariance, which is the sum of the measurement covariance matrix, R(k+1), and the propagated state covariance transformed through the measurement sensitivity matrix, $H\left(k+1\right)P\left(k+1|k\right)H{\left(k+1\right)}^{T}$. In the absence of multipath tracking errors, the resulting NIS statistic is chi-squared distributed with $n_{z}$ degrees of freedom. If the NIS statistic exceeds a chosen threshold, then the signal with the largest normalized innovation is dropped. This continues until the NIS statistic falls below the threshold or the number of remaining signals drops to a preset minimum number of required signals.

##### Simulation Results

**Figure 5:**
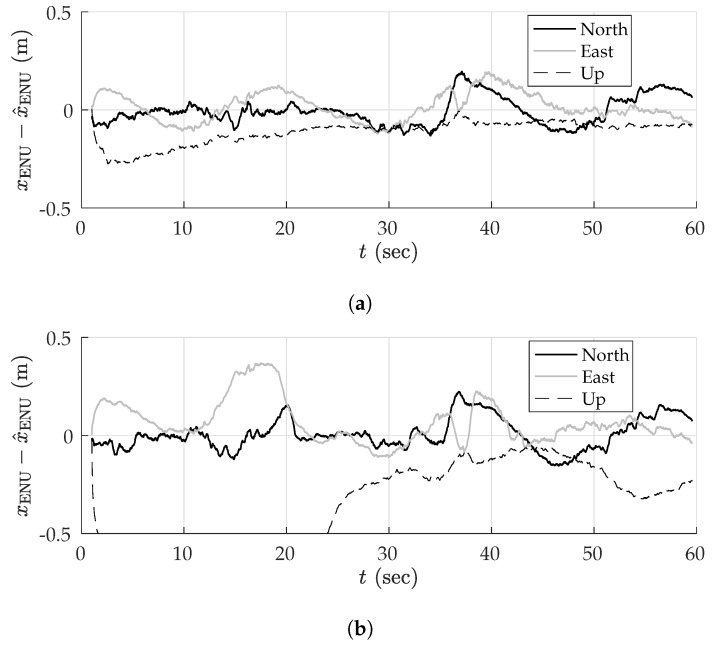
Mean position error in the east-north-up (ENU) frame over 1000 sessions due to multipath. (**a**) Ideal multipath exclusion. (**b**) Normalized innovation statistic (NIS)-based multipath exclusion. The black, gray, and dashed-black lines represent the error in the east, north, and up directions, respectively. The up error in the bottom panel reached a maximum magnitude of 1.75 m.

Figure 5 shows the mean position error in the east, north, and up directions over 1000 sessions for the two multipath rejection schemes mentioned previously. From Figure 5a, it can be seen that sub-20 cm average error is achievable with hypothetical ideal multipath exclusion. A closer look at Figure 4 and Figure 5a reveals that the decimeter-level sinusoidal position error trend, initially in the north direction and later in the east direction, in fact corresponds with the along-track accelerations of the vehicle that were not adequately tracked by the nearly-constant-velocity-model-based navigation filter.

Figure 5b shows that the NIS test based exclusion of signals was able to approach the performance of ideal exclusion in the horizontal plane, save for the first stationary period where the vehicle was moving at low speed between buildings on both sides. The average vertical position error was much worse, growing as large as 1.75 m in magnitude.

To determine whether the average errors shown in Figure 5 are in fact persistent biases, a study of the standard deviation of position errors was conducted. The standard deviation of the average errors in east, north, and up directions was computed for disjoint averaging ensembles of size 1, 2, 4, 8, 16, 32, 50, and 100 sessions taken from the total of 1000 simulated sessions. For instance, 125 disjoint ensembles of eight sessions were selected, and the position errors were averaged over the eight sessions in each set. The standard deviation of the eight-session-averaged errors was then computed across the 125 ensembles. In the case of an averaging ensemble with only a single session (i.e., no averaging), the computed standard deviation is simply the measured standard deviation of the position error across all 1000 simulated runs. In the case of averaging over 100 sessions, the standard deviation is computed based on 10 disjoint averaging ensembles of 100 sessions each.

**Figure 6:**
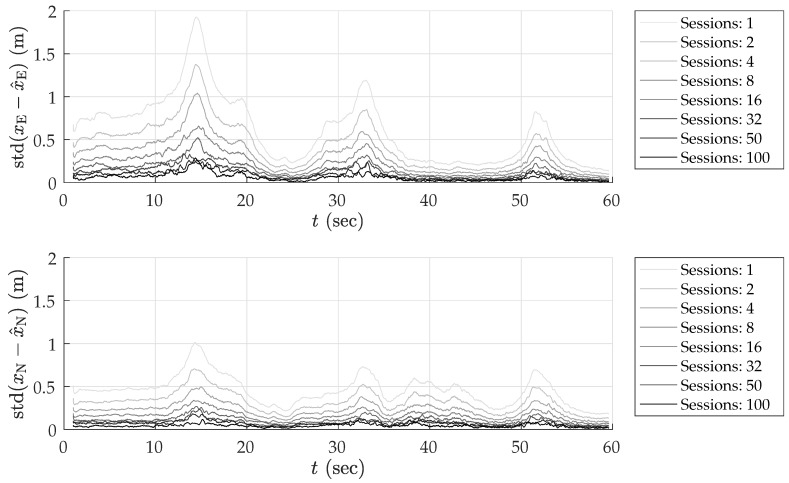
Standard deviation of average position error in east and north directions for NIS-based multipath exclusion as a function of the number of sessions over which the errors are averaged. **Top panel**: standard deviation in the east direction. **Bottom panel**: standard deviation in the north direction.

Note that because the simulation study was based on the same 1000 simulations for all averaging ensembles, the east, north, and up means taken across all averaging ensembles are equivalent to those shown in Figure 5. The more interesting trend is the decreasing standard deviation with increasing size of the averaging ensemble, as shown in Figure 6 for the case of NIS-based multipath rejection and for the east and north error components. As expected, the standard deviation of errors was higher at locations where the vehicle moved at low speed and multipath decorrelated slowly. Additionally, the standard deviation was larger at the beginning of the trajectory where the street was lined with tall buildings on both sides.

The standard deviation of the average east and north position error over 100 sessions was bounded below 15–20 cm. Thus, it is highly likely that the ∼40-cm error in the north direction between 15–20 s in Figure 5b is in fact a persistent non-zero bias.

Table 3 summarizes the results of the multipath simulation study. It shows the 95-percentile horizontal error magnitude for increasing averaging ensemble sizes and for both ideal and NIS-based multipath exclusion. The 0–60 s average case lists the 95-percentile error over the entire trajectory, whereas the 13–19 s average case lists the 95-percentile error in the worst-case segment of the trajectory in terms of horizontal position bias and standard deviation. This challenging segment is illustrative of persistent problem spots that will arise in urban areas, within which multipath-induced biases will be larger than average. As expected, the 95-percentile error in Table 3 shrank as the averaging ensemble size became larger. For the urban corridor and vehicle dynamics considered in this simulation, NIS-based exclusion achieved 35 cm 95-percentile horizontal error with averaging over 100 sessions. Even in the worst-case region of the trajectory, the 95-percentile horizontal error remained below 50 cm. As multipath exclusion approaches the ideal case, with aid from other sensors or a 3D model of the surroundings, for example, the 95-percentile horizontal error could be reduced to as low as 25 cm for the simulated corridor.

From the Section 3.3.3’s analysis of asymptotic ionospheric errors, and from this section’s multipath simulation study, one can draw the following conclusion: so long as the asymptotic horizontal position errors of the ionosphere corrections are below 5 cm, as is true for the Fast-PPP model, and assuming statistical independence of ionospheric and multipath errors, it appears feasible to achieve 50-cm horizontal positioning accuracy at approximately 95% by averaging over 100 mapping sessions.

## 4. Globally-Referenced Electro-Optical SLAM (GEOSLAM)

This section describes a simultaneous localization and mapping (SLAM) pipeline capable of globally-referenced collaborative multi-session digital mapping. The pipeline combines visual measurements from a stereo visible-light camera system with position measurements from GNSS signals. The objective of this pipeline is to demonstrate the development of an accurate digital map based on multiple mapping sessions with standard GNSS position estimates. The following sections detail the visual SLAM pipeline, the integration of GNSS measurements, and the techniques for multi-session mapping developed in GEOSLAM.

### 4.1. Visual SLAM

The visual SLAM component of GEOSLAM is similar to existing high-performance SLAM pipelines developed in the robotics community [54,55,56]. Visual SLAM algorithms may be categorized as either sparse or dense. Sparse visual SLAM algorithms [54,55] create a map of distinctive features such as corners or edges in the scene, while dense SLAM algorithms [56] map the depth for each pixel in the captured frames. The point cloud generated by sparse SLAM algorithms is sufficient for the purpose of localization. Dense reconstruction is appealing to the human eye, but does not provide any tangible benefit to localization, while consuming much more computational resources. As a result, GEOSLAM implements sparse feature-point-based SLAM.

In [57] it was shown that for the visual SLAM problem, structure-from-motion BA (batch non-linear optimization) outperforms filtering techniques such as the extended Kalman filter, yielding higher accuracy per unit of computing time. It was also noted that having a high number of features points per image frame provides better accuracy than having a large number of frames with fewer feature points per frame. Thus, in typical practice, only a select subset of frames among those captured is retained for processing; frames in this subset are called keyframes. Most recent state-of-the-art visual SLAM algorithms use a keyframe-based BA approach instead of sequential filtering. Likewise, GEOSLAM performs BA-based non-linear optimization to refine both structure and motion.

**Figure 7:**
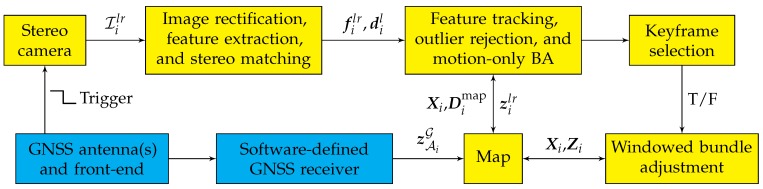
Globally-referenced electro-optical simultaneous localization and mapping (GEOSLAM) block diagram. BA: bundle adjustment.

Figure 7 shows a block diagram representation of the system architecture proposed in this paper. The yellow-colored blocks in this figure are components of the GEOSLAM pipeline, detailed next.

By way of notation, let

$$z_{nm}^{lr}≜\left(u_{nm}^{l},v_{nm}^{l},u_{nm}^{r}\right)$$

denote the image plane location of the stereo-matched feature matched to the *m*th map point, $p_{m}$, in the *n*th stereo keyframe, $K_{n}$. The horizontal and vertical coordinates are denoted *u* and *v*, respectively, while the superscripts *l* and *r* denote the left and right camera frames, respectively. Note that the feature location is specified by only three coordinates. The vertical feature coordinate in the right camera frame, $v_{nm}^{r}$, is omitted because for an undistorted and rectified camera model it must hold that $v_{nm}^{l}=v_{nm}^{r}$, making one of the coordinates redundant. If $p_{m}$ is not matched to any feature in $K_{n}$, then let $z_{nm}^{lr}=⌀$. Furthermore, let

$$M_{n}=\left\{m\;:\;z_{nm}^{lr}\neq ⌀\right\}$$

denote the set of indices of all map points matched to some feature in $K_{n}$. In the visual SLAM literature, the covisibility window of keyframe $K_{i}$ is defined as the set of keyframes that share at least *T* map points with $K_{i}$. Mathematically, the covisibility window of keyframe $K_{i}$ is the set of keyframes with indices

$$\mathrm{cov}(i)≜\left\{n\;:\;|M_{n}\cap M_{i}|>T\right\},$$

where |A| denotes the cardinality of the set A. The covisibility window determines the keyframes to be optimized in a windowed BA. The visibility of common points is regarded as a proxy for correlation between the structure-from-motion states. However, in a sensor fusion architecture, the states for other sensors (e.g., GNSS) may be spatially correlated beyond the covisibility window. Furthermore, other sensors may experience outages that extend beyond the covisibility window. In such a scenario, it would be desirable to optimize over a batch of keyframes that span the availability gap. Accordingly, GEOSLAM extends the concept of covisibility to *N* levels as

$$\mathrm{cov}(i,N)≜\left\{\begin{array}{cc} \left\{n\;:\;|M_{n}\cap \left(\cup_{k\in \mathrm{cov}(i,N-1)}M_{k}\right)|>T\right\} & N>1,\\ \left\{n\;:\;|M_{n}\cap M_{i}|>T\right\} & N=1. \end{array}\right.$$

When processing $K_{i}$, GEOSLAM’s objective is to estimate the map point 3D locations $x_{p_{m}}^{S}\in R^{3}$ and keyframe poses $\left(x_{C_{n}}^{S},\theta_{C_{n}}^{S}\right)\in \left(R^{3},R^{3}\right)$ in the *N*-level covisibility window for the *i*th keyframe, where S stands for the local SLAM frame, $C_{n}$ is the left camera coordinate frame associated with $K_{n}$, and $\theta_{C_{n}}^{S}$ is the angle-axis representation of the keyframe orientation. The state vector to be estimated is represented as

(4) $$X_{i}≜{\left[\left\{\left(x_{C_{n}}^{S},\theta_{C_{n}}^{S}\right)\;:\;n\in \mathrm{cov}(i,N)\right\},\left\{x_{p_{m}}^{S}\;:\;m\in \cup_{k\in \mathrm{cov}(i,N)}M_{k}\right\}\right]}^{T},$$

where the two sets on the right-hand side are arranged as a concatenation of row vectors so that $X_{i}$ becomes a column vector.

When triggered by the GNSS front end, the camera setup captures a pair, denoted $I_{i}^{lr}$, of concurrent images from the left and right cameras, where the subscript denotes that the current pair is a candidate to be the *i*th stereo keyframe $K_{i}$. The intrinsic and extrinsic parameters of the stereo camera setup are assumed to have been calibrated a priori. The stereo image pair is then undistorted and rectified according to the given calibration, and SIFT features are detected and computed separately for each image [58]. SIFT feature matching is performed between the left and right image with the additional constraint that matching features must have approximately the same vertical coordinate to within a few pixels. The set of stereo feature measurements for $I_{i}^{lr}$, $f_{i}^{lr}$, and the set of feature descriptors as computed in the left image, $d_{i}^{l}$, are passed on to the tracking module.

The tracking module has access to the 3D map point positions within $X_{i}$ and to the set of SIFT descriptors, $D_{i}^{\mathrm{map}}$, corresponding to the map points expected to be seen in the candidate keyframe $I_{i}^{lr}$. The tracker performs directed matching of the features between the stereo image and the map. First, a quick feature matching is performed using the Fast Approximate Nearest Neighbor Search Library (FLANN) [59]. With sufficient matches, an initial approximation of the current camera pose is obtained using the five-point algorithm wrapped in random sample consensus (RANSAC) iterations. With this approximate pose, an iteration of exhaustive nearest neighbor search is performed for each map point potentially in view of the camera, but only within a small window of its projected position on the image plane. Subsequently, RANSAC iterations are performed on the full set of feature matches to remove any remaining outliers, and a motion-only BA is performed wherein the current camera pose is optimized based on the feature matches to a fixed set of 3D map points.

After tracking the stereo image pair as described above, GEOSLAM decides whether or not the candidate keyframe $I_{i}^{lr}$ must be selected as a keyframe. This decision is made based on the number of map points that were matched to the image features, and the distance traveled by the platform since the last keyframe was chosen. New keyframes are not spawned if the platform is nearly stationary. If the platform is in motion, and the number of feature matches to the map drops below a threshold, then the candidate keyframe $I_{i}^{lr}$ is chosen as keyframe $K_{i}$, windowed BA is performed over *N* levels of covisibility, and the unmatched stereo features in $K_{i}$ are spawned as new map points. Additionally, if $I_{i}^{lr}$ is selected to be $K_{i}$, then the set of measurements from the features matched to the map, denoted $z_{i}^{lr}≜\left\{z_{im}^{lr}\;:\;m\in M_{i}\right\}$, along with their SIFT descriptors, are passed on to the map module for storage, future feature matching, and processing in the windowed BA routine.

In a visual-only SLAM system, the state vector $X_{i}$ is optimized with respect to the measurement vector $Z_{i}$, defined as

$$Z_{i}≜{\left[\left\{z_{n}^{lr}\;:\;n\in \mathrm{cov}\left(i,N\right)\right\}\right]}^{T}.$$

The windowed BA routine in GEOSLAM minimizes the 3D-to-2D reprojection error. The error term $e_{nm}$ for observation of map point $p_{m}$ in the stereo keyframe $K_{n}$ is given as

$$e_{nm}=z_{nm}^{lr}-\Pi \left(x_{p_{m}}^{S},x_{C_{n}}^{S},\theta_{C_{n}}^{S}\right),$$

where Π is the projection function for an undistorted and rectified stereo camera

$$\begin{array}{cc} \Pi \left(x_{p_{m}}^{S},x_{C_{n}}^{S},\theta_{C_{n}}^{S}\right) & =\left[\begin{array}{c} f\frac{x_{nm}}{z_{nm}}+c_{u}\\ f\frac{y_{nm}}{z_{nm}}+c_{v}\\ f\frac{x_{nm}}{z_{nm}}+c_{u}-bf \end{array}\right],\\ {\left[x_{nm},y_{nm},z_{nm}\right]}^{T} & =R{\left(\theta_{C_{n}}^{S}\right)}^{T}\left(x_{p_{m}}^{S}-x_{C_{n}}^{S}\right), \end{array}$$

in which R(·) denotes the rotation matrix corresponding to the argument angle-axis vector, and *f*, $\left(c_{u},c_{v}\right)$, and *b* are the focal length, the principal point, and the baseline distance between the left and right cameras of the rectified stereo camera model, respectively. The cost function to be minimized for visual SLAM is given as

$$C_{i}=\sum_{n\in \mathrm{cov}(i,N)}\sum_{m\in M_{n}}\rho \left(e_{nm}^{T}\Omega_{nm}^{-1}e_{nm}\right),$$

where ρ may be the standard least squares cost function ρ(·)=(·) or a more robust cost function such as the Huber or Tukey cost functions, and where $\Omega_{nm}=\sigma_{nm}^{2}I_{3\times 3}$ is the covariance of the feature measurements.

GEOSLAM performs BA minimization via Google’s ceres-solver. The automatic differentiation feature of ceres-solver is used to compute the Jacobian for the measurement model.

An important feature of the GEOSLAM visual pipeline is the ability to merge maps from multiple mapping sessions. This is embodied in an algorithm similar to the loop closure technique from the visual SLAM literature [60]. Map merging is described in detail in Section 4.3.2.

### 4.2. GNSS Aiding

Conventional visual SLAM algorithms are known to drift from the true platform trajectory as a function of the distance traveled by the platform. Furthermore, the map of the structure is created in the arbitrary S frame. Such a map cannot be intelligibly shared with another mapping agent having a different S frame. Meanwhile, GNSS position estimates are obtained in the global G frame and do not exhibit any distance-dependent drift. Accordingly, GEOSLAM ingests standard GNSS position estimates from a software-defined GNSS receiver, called GRID/pprx [6,61], in a tightly-coupled architecture to create a globally-referenced map, enable cooperative multi-session mapping, and constrain the drift of visual SLAM. Since the stereo camera setup is triggered by the same clock that drives digitization of the GNSS samples (see Figure 7), it is possible to produce GNSS measurements synchronized with the camera image epochs. This section details the various coordinate frames in GNSS-aided visual SLAM, the updated BA cost function, and an initialization routine required to enable GNSS aiding in SLAM.

#### 4.2.1. Coordinate Frames

The GNSS-aided visual SLAM system has three coordinate frames of interest: the $K_{i}$ camera frame $C_{i}$, the local SLAM frame S, and the global frame G. The SLAM frame S adopts the position and orientation of the first keyframe prior to optimization as its origin and orientation. Thus, S is fixed relative to G, but each $C_{i}$ changes relative to G as the platform moves.

Note that the structure-from-motion states in Equation (4) are represented in the S frame, whereas the $K_{n}$th keyframe’s corresponding GNSS measurement, denoted $z_{A_{n}}^{G}\in R^{3}$, is natively represented in the G frame. The latter is transformed to the S frame through an unknown but fixed rotation, $R_{G}^{S}\in SO\left(3\right)$, and translation, $t_{G}^{S}\in R^{3}$. This transformation is estimated at initialization as explained in Section 4.2.2. After initialization, $z_{A_{n}}^{G}$ is rendered in S as

$$z_{A_{n}}^{S}=R_{G}^{S}z_{A_{n}}^{G}+t_{G}^{S}.$$

GEOSLAM estimates the 6-DoF pose of the left camera, but the GNSS antenna phase center is not co-located with the camera center; rather, it is offset from the camera center by a fixed vector (same for all *n*) in $C_{n}$ denoted, $t_{A_{n}}^{C_{n}}\in R^{3}$. Thus, the error term associated with the GNSS position estimate for $K_{n}$ is given as

$$e_{A_{n}}=z_{A_{n}}^{S}-\left(x_{C_{n}}^{S}+R\left(\theta_{C_{n}}^{S}\right)t_{A_{n}}^{C_{n}}\right).$$

Under the assumption of temporally-uncorrelated GNSS errors, the updated BA cost function to be minimized is

$$C_{i}=\sum_{n\in \mathrm{cov}(i,N)}\left[\sum_{m\in M_{n}}\rho \left(e_{nm}^{T}\Omega_{nm}^{-1}e_{nm}\right)+e_{A_{n}}^{T}\Gamma_{n}^{-1}e_{A_{n}}\right],$$

where $\Gamma_{n}=R_{G}^{S}\Gamma_{n}^{\prime }{\left(R_{G}^{S}\right)}^{T}$ and $\Gamma_{n}^{\prime }$ is the covariance matrix of the GNSS position estimate associated with $K_{n}$, expressed in G.

#### 4.2.2. Initialization in GNSS-Aided SLAM

When initializing, GEOSLAM performs visual-only SLAM for the first $N_{i}$ keyframes in the S frame, and stores the GNSS position measurements of the antenna provided in the G frame. Subsequently, GEOSLAM finds the least-squares Euclidean transformation to obtain the optimal rotation matrix $R_{G}^{S}$ and translation vector $t_{G}^{S}$ between the two coordinate systems from the set of vector observations. Note that a full similarity transformation is not required since the known stereo baseline renders the S frame with correct scaling. The estimated Euclidean transformation minimizes the squared difference between the transformed GNSS measurements in S and the visual SLAM predicted trajectory of the GNSS antenna, also in S. The specific method used to estimate the transformation is based on SVD decomposition as discussed in [62].

(5) $$\left(R_{G}^{S},t_{G}^{S}\right)=\underset{R\in SO\left(3\right),t\in R^{3}}{arg min}\sum_{n=1}^{N_{i}}{∥\left(Rz_{A_{n}}^{G}+t\right)-\left(x_{C_{n}}^{S}+R\left(\theta_{C_{n}}^{S}\right)t_{A_{n}}^{C_{n}}\right)∥}^{2}$$

It must be noted that this transformation need only be approximately correct such that the GNSS estimates, used as measurements, will not diverge with respect to the visually-derived trajectory. Because the jointly estimated trajectory in S gets transformed back to G using the same approximate transformation, any errors in the transformation are cancelled.

### 4.3. Multi-Session Mapping

Refinement of the visual feature map over multiple sessions with time-separated GNSS measurements is central to the idea of approaching the accuracy limit of mapping with standard GNSS. Consider a vehicle revisiting an area mapped previously in one or more sessions. When GEOSLAM matches greater than *T* features in the current keyframe to the features already present in the prior map, the keyframes from the previous sessions in that section of the map are included in the covisibility window of the current keyframe. After such a merge is detected and verified, a BA may be performed on the covisible keyframes from multiple sessions to average time-separated standard GNSS errors. It is important to note that multi-session mapping can only be realized when sufficient feature matches are found between multiple sessions. This is not a straightforward task, as evidenced by recent efforts on lifelong feature mapping efforts [63]. This issue is further discussed in Section 5. In the current section, multi-session map database management and map merging are discussed.

#### 4.3.1. Map Database

Storage and reuse of maps is a pre-requisite for multi-session mapping. For a given session, the SLAM map is created in the S frame. However, such a map is not readily usable in successive mapping sessions since the S frame is distinct for each session. Fortunately, the integration of visual SLAM with GNSS enables transformation of the SLAM map in to the G frame.

At the end of the *p*th mapping session in the local frame $S_{p}$, GEOSLAM stores the data to a map database after applying the $\left(R_{G}^{S_{p}},t_{G}^{S_{p}}\right)$ transformation, as estimated during initialization for the *p*th session, to all map point positions, all keyframe poses, and all GNSS measurements associated with each keyframe:

$$\begin{array}{cc} x_{C_{n}}^{G} & ={\left(R_{G}^{S_{p}}\right)}^{T}\left(x_{C_{n}}^{S_{p}}-t_{G}^{S_{p}}\right);\;R\left(\theta_{C_{n}}^{G}\right)={\left(R_{G}^{S_{p}}\right)}^{T}R\left(\theta_{C_{n}}^{S_{p}}\right),\\ x_{p_{m}}^{G} & ={\left(R_{G}^{S_{p}}\right)}^{T}\left(x_{p_{m}}^{S_{p}}-t_{G}^{S_{p}}\right),\\ z_{C_{n}}^{G} & ={\left(R_{G}^{S_{p}}\right)}^{T}\left(z_{C_{n}}^{S_{p}}-t_{G}^{S_{p}}\right). \end{array}$$

At the beginning of the (p+1)th session, GEOSLAM again estimates the $\left(R_{G}^{S_{p+1}},t_{G}^{S_{p+1}}\right)$ transformation during initialization. The map database from previous session(s) is then loaded after applying the transformation for the p+1 session, such that the prior map points, keyframes, and measurements are rendered in the $S_{p+1}$ frame:

$$\begin{array}{cc} x_{C_{n}}^{S_{p+1}} & =R_{G}^{S_{p+1}}x_{C_{n}}^{G}+t_{G}^{S_{p+1}};\;R\left(\theta_{C_{n}}^{S_{p+1}}\right)=\left(R_{G}^{S_{p+1}}\right)R\left(\theta_{C_{n}}^{G}\right),\\ x_{p_{m}}^{S_{p+1}} & =R_{G}^{S_{p+1}}x_{p_{m}}^{G}+t_{G}^{S_{p+1}},\\ z_{C_{n}}^{S_{p+1}} & =R_{G}^{S_{p+1}}z_{C_{n}}^{G}+t_{G}^{S_{p+1}}. \end{array}$$

After loading the prior map, the standard GEOSLAM pipeline is executed for each stereo image pair in the (p+1)th session. In addition, GEOSLAM attempts to detect if the vehicle is currently passing through a previously-mapped region. If so, a map merge is declared and the current and prior keyframes are jointly optimized, as detailed in Section 4.3.2. Finally, at the end of the mapping session, the combined map is stored back in the database as described before.

#### 4.3.2. Map Merging

As mentioned before, the matching of feature points across multiple sessions is central to the idea of averaging standard GNSS errors. Once sufficiently many features are matched between the current stereo keyframes and prior map points, GEOSLAM declares a map merging event. This is akin to the well-known problem of detecting loop closure in the visual SLAM literature [60]. This section details GEOSLAM’s map merging and loop closing routine. Hereafter, the terms map merging and loop closure are used interchangeably since GEOSLAM treats them identically.

First, note that when detecting a map merge event, feature matching must be attempted against map points that have not been matched in the most recent keyframes. Thus, after processing the *i*th keyframe, a possible merge is checked for against the set of map points $\left\{m\;:\;m\notin \cup_{n\in \mathrm{cov}(i,N)}M_{n}\right\}$. If this bag-of-words-style feature matching succeeds, then RANSAC iterations are performed to determine whether the matches are geometrically consistent, as well as to robustly estimate the camera pose $\left({\overset{ˇ}{x}}_{C_{i}}^{S},{\overset{ˇ}{\theta }}_{C_{i}}^{S}\right)$ implied by the merge event. If enough inliers are found, the map merge routine is executed.

**Figure 8:**
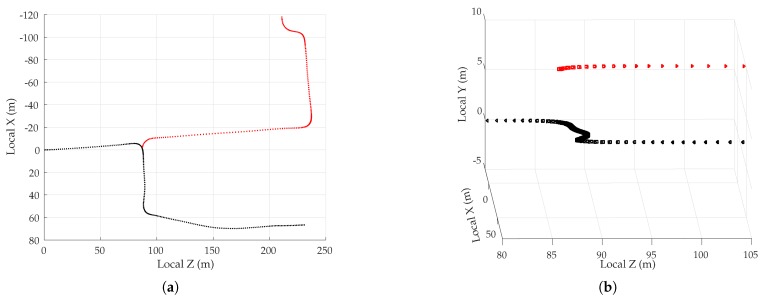
GEOSLAM trajectories at the instant when a merge has been detected and verified. The cameras colored black are keyframes from a prior map, and those colored red are from the current session. (**a**) Top view of the trajectories. (**b**) View from 5${}^{\circ }$ elevation showing a discontinuity in the vertical component.

**Figure 9:**
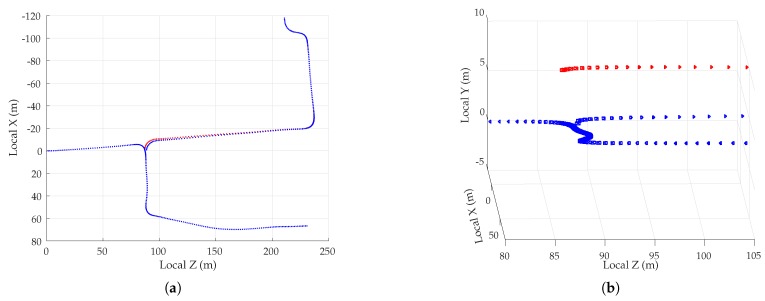
GEOSLAM trajectories post-pose-graph optimization (in blue), overlaid on the corresponding (black and red) trajectories from Figure 8. All keyframes are colored blue at this stage since prior and current keyframes are now connected. (**a**) Top view of the trajectories. Note that the discontinuity at the merge location is smoothly distributed across $N_{m}$ levels of covisibility in the current session, and that the keyframe poses from the prior map are unchanged at this stage. (**b**) View from 5${}^{\circ }$ elevation. Keyframes from the current trajectory have been adjusted to remove the discontinuity, blue and black keyframes exactly overlap. Not shown: the corresponding map points in the current session are also adjusted to match the updated keyframe poses.

**Figure 10:**
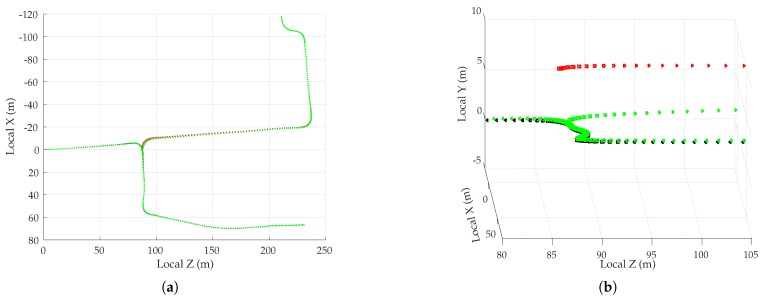
GEOSLAM trajectories after joint BA of current and prior keyframes (in green), overlaid on the corresponding (black and red) trajectories from Figure 8. (**a**) Top view of the trajectories. Note that both the current and prior keyframes (and map points, not shown) have been adjusted to optimally minimize the BA cost function over $N_{m}$ levels of covisibility. (**b**) View from 5${}^{\circ }$ elevation.

The map merging process is depicted visually in Figure 8, Figure 9 and Figure 10. A typical merge situation is shown in Figure 8, where the platform pose at the *i*th keyframe is inconsistent with the prior map at the merge location. To avoid such discontinuity in the ensuing joint BA, as an initial guess GEOSLAM enforces that the *i*th keyframe pose be consistent with the pose implied by the visual merge matches $\left({\overset{ˇ}{x}}_{C_{i}}^{S},{\overset{ˇ}{\theta }}_{C_{i}}^{S}\right)$, and that the keyframes and map points from the prior session(s) be unchanged. To this end, a pose-graph optimization [64] is performed over a large $N_{m}$-level covisibility window for $K_{i}$, where the relative translations and rotations between covisible keyframes, as estimated in the current session, are provided as delta-pose measurements, while the 6-DoF poses for the terminal nodes in the covisibility window, as well as for $K_{i}$, are held constant. In particular, let $K_{0}$ denote the set of terminal keyframes in the covisibility graph $\mathrm{cov}(i,N_{m})$. Furthermore, define the delta-pose pseudo-measurements

$$\begin{array}{cc} \delta x_{nk}^{S} & ≜{\hat{x}}_{C_{n}}^{S}-{\hat{x}}_{C_{k}}^{S},\\ \delta \theta_{nk}^{S} & ≜\theta \left(R\left({\hat{\theta }}_{C_{n}}^{S}\right)R{\left({\hat{\theta }}_{C_{k}}^{S}\right)}^{T}\right), \end{array}$$

where the superscript $\left(\hat{\cdot }\right)$ denotes GEOSLAM’s estimate of the state before the merge event, and θ(·) denotes the angle-axis representation of the input rotation matrix. The pose-graph optimization minimizes the following cost function with respect to $\left(x_{C_{n}}^{S},\theta_{C_{n}}^{S}\right)\forall n\in \mathrm{cov}\left(i,N_{m}\right)$:

$$C=\sum_{n\in \mathrm{cov}(i,N_{m})}\sum_{k\in \mathrm{cov}(n,1)}\left[{∥\left(x_{C_{n}}^{S}-x_{C_{k}}^{S}\right)-\delta x_{nk}^{S}∥}_{P_{\delta x}^{-1}}^{2}+{∥\theta \left(R\left(\theta_{C_{n}}^{S}\right)R{\left(\theta_{C_{nk}}^{S}\right)}^{T}\right)-\delta \theta_{n-1}^{S}∥}_{P_{\delta \theta }^{-1}}^{2}\right],$$

where ${∥q∥}_{P}^{2}=q^{T}Pq$, with the following constraints:

$$\begin{array}{cc} \left(x_{C_{i}}^{S},\theta_{C_{i}}^{S}\right) & =\left({\overset{ˇ}{x}}_{C_{i}}^{S},{\overset{ˇ}{\theta }}_{C_{i}}^{S}\right),\\ \left(x_{C_{n}}^{S},\theta_{C_{n}}^{S}\right) & =\left({\hat{x}}_{C_{n}}^{S},{\hat{\theta }}_{C_{n}}^{S}\right)\forall n\in K_{0}. \end{array}$$

As a result of this pose-graph optimization, any discontinuity at the merge location between the prior and current keyframes is smoothed out, as shown in Figure 9. Subsequently, the map points as seen in the current keyframes are also adjusted in accordance to the pose-graph optimization. Finally, the duplicated map points near the merge location are fused together. As a result of shared feature matches between the current and prior keyframes, the updated covisibility window for $K_{i}$ includes keyframes from prior session(s).

The merged map is then optimized in a windowed BA, this time with feature point coordinates and GNSS positions as measurements. Note that this is a joint windowed BA with both current and prior keyframes and map points. As a result, both the current and prior states are appropriately adjusted based on the number and covariance of the feature point and GNSS measurements. The result of the map merging routine is shown in Figure 10.

## 5. Empirical Results

To validate the results obtained in the above analyses, GNSS and visual data were collected in a moderate urban area north of the University of Texas at Austin campus in Austin, TX. This section presents the data collection setup, error statistics of various flavors of code-phase GNSS positioning, and results from GEOSLAM’s multi-session GNSS-aided-visual mapping.

### 5.1. Rover and Reference Platforms

The rover GNSS receiver is one among several sensors housed in an integrated perception platform called the University of Texas Sensorium [6]. Designed for connected and automated vehicle research, the Sensorium is a self-contained sensor housing that can be mounted atop any standard passenger vehicle. Two Antcom G8Ant-3A4TNB1 triple-frequency patch antennas are flush-mounted in the cross-track direction on the Sensorium’s upper plate, separated by just over one meter. The antennas’ signals are routed to a unified radio frequency (RF) front end whose output intermediate frequency (IF) samples are processed in real-time (to within less than 10 ms latency) by the Sensorium’s onboard computer. The samples are also stored to disk for post-processing. The experimental setup also includes a surveyed GNSS reference station that aids in the generation of the ground truth trajectory.

The GNSS data were processed by a software-defined GNSS receiver tracking signals from GPS L1 C/A, GPS L2CLM, Galileo E1, and SBAS. Data from both the primary (passenger’s side) and secondary (driver’s side) antennas were used to reconstruct a sub-dm-accurate CDGNSS-based ground truth trajectory, as described in [6]. Enhanced code-phase positioning was performed on the data from the primary antenna, incorporating precise orbit and clock products from IGS, ionospheric corrections from WAAS satellites, and the Saastamoinen model for tropospheric corrections, in addition to NIS-based exclusion of multipath signals. Double-differenced pseudorange-based positioning was also performed with the data from the primary antenna, as discussed later in this section. The code-phase-based position estimates were (i) compared against the ground truth from the primary antenna to study the code-phase positioning error statistics, and (ii) fed to GEOSLAM for vision-GNSS sensor fusion. The primary antenna feed was also input to a ublox M8T receiver for comparison against the enhanced code-phase software receiver.

The Sensorium features a front-facing stereo camera rig composed of two Basler acA2040-35gm cameras that capture synchronous stereo image pairs when triggered by a signal tied to the GNSS front-end’s sampling clock. The images are captured in grayscale at 10 frames per second and timestamped by the Sensorium’s computer. The cameras are configured to automatically adjust the exposure time based on lighting, while the focal length, focus, and aperture are held fixed, having been adjusted physically prior to capture.

### 5.2. Test Route

**Figure 11:**
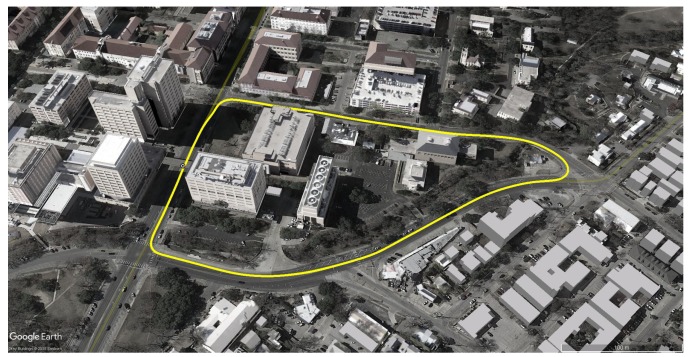
An overview of the 1-km test route. The Dean Keeton corridor, toward the left, is spanned by a pedestrian bridge and flanked by buildings on both sides. A total of 75 laps of the test route were driven over six separate campaigns.

The test route was a 1-km loop north of the University of Texas at Austin campus in Austin, TX. The route included a variety of light-to-moderate urban conditions, from open-sky to overhanging trees to built-up areas. The Dean Keeton corridor, toward the left in Figure 11, was the most challenging stretch along the test route for GNSS positioning. It passes below a pedestrian bridge and is flanked on both sides by buildings ranging from 30 to 65 meters tall set back 28 meters from the center of the roadway.

To study the code-phase-based positioning error characteristics over time-separated sessions in the same area, and to perform multi-session mapping with GEOSLAM, multiple laps of the test route were driven over six separate campaigns. The first two campaigns were conducted on 21 December 2017 and 15 January 2018, while the other four campaigns were conducted in pairs of two on 3 June 2018 and 4 June 2018. The GNSS error charts are presented for a total of 75 laps of the test route, while multi-session mapping with GEOSLAM was performed over eight laps/sessions of data from the four latest campaigns.

**Figure 12:**
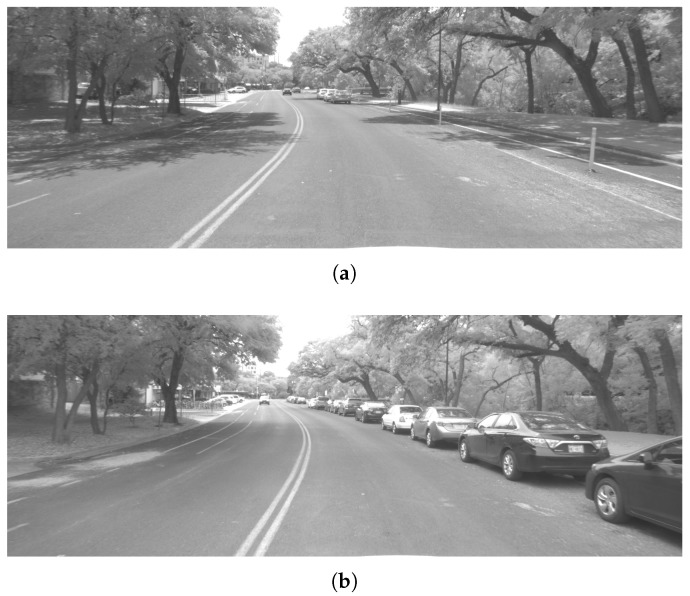
Different visual conditions on two days of data collection. (**a**) An image captured on the first day of data collection. Note the sharp shadows and absence of parked cars. (**b**) An image captured on the second day of data collection. Note the absence of sharp shadows and complete blockage of curb due to parked cars.

Imagery collected over the four June 2018 campaigns exhibits appreciable visual diversity, offering a real-world challenge to multi-session GEOSLAM operation. Figure 12a,b show the variation in lighting and visual features between the data collected on 3 June 2018 and 4 June 2018.

### 5.3. Empirical GNSS Error Analysis

**Figure 13:**
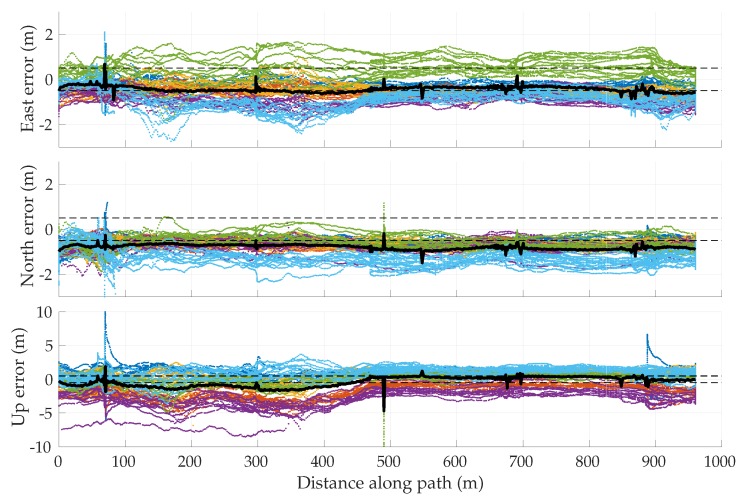
Errors in enhanced code-phase position estimates with respect to ground truth in the east, north, and up directions. Different colors distinguish data from six different campaigns. The dashed reference lines are drawn at ± 50 cm. The solid black lines show the mean positioning error over the six campaigns. The error standard deviation is nearly constant along the path in the horizontal plane at ∼0.6 m in the east and ≈0.4 m in the north direction. In the up direction, the standard deviation is ∼2.1 m for the first 400 m along the path, and ≈1.3 m for the rest.

**Figure 14:**
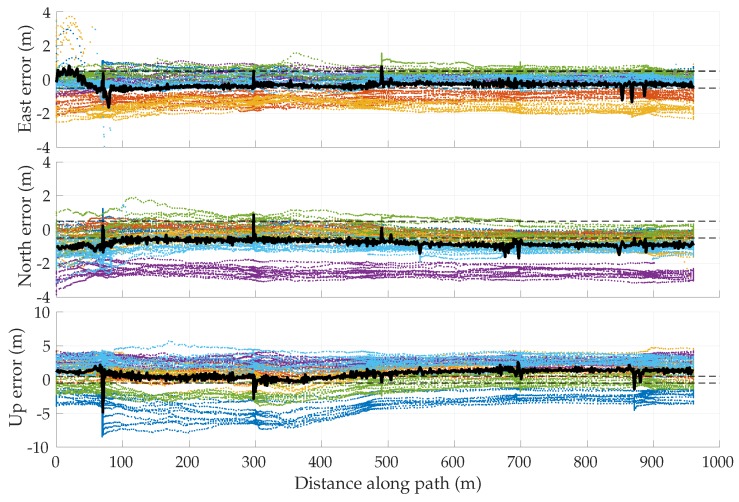
Errors in ublox M8T position estimates with respect to ground truth in the east, north, and up directions. Different colors distinguish data from six different campaigns. Dashed reference lines are drawn at ± 50 cm. The solid black lines show the mean positioning error over the six campaigns. The error standard deviation in the east is ∼1.5 m over the first 100 m along the path and ∼0.7 m over the rest; ∼0.9 m in the north; and ∼2.7 m over the first 400 m and ∼2 m over the rest in the up direction.

Figure 13 shows the error in the enhanced code-phase GNSS position solutions with respect to the ground truth. The error is plotted versus the distance along the 1-km loop. The beginning of this loop was taken to be immediately after the overhead pedestrian bridge along the Dean Keeton corridor. It is observed that the enhanced code-phase GNSS errors are clustered separately for each of the campaigns, and that each cluster is offset from zero by as much as 1 m in the horizontal plane. Such error characteristics are representative of ionospheric modeling errors, which have a long decorrelation time. It is also evident that the error variance was larger as the receiver exits the challenging portion of the loop at which point the tracking loops were recovering from signal loss under the bridge. The effect was especially pronounced in the vertical direction. Figure 14 shows similar errors for the commercial ublox M8T receiver. The error traces from the ublox receiver show a wider spread than the enhanced code-phase receiver, likely due to lack of precise orbit and clock corrections.

On the basis of Figure 13 and Figure 14, one might be tempted to conclude that errors in enhanced code-phase and stand-alone GNSS navigation solutions are substantially non-zero-mean, especially in the north and up directions, despite the overhead GNSS constellation changing substantially between sessions. It certainly appears that the permanent structures (buildings, bridge) along the test loop left a bias in the vertical direction during the first 400 m along the loop. However, the bias in the north direction, and to a lesser extent in the east, may only be an artifact of the small sample size: ionospheric modeling errors were not yet averaged down to nearly zero in the east and ∼30 cm in the north, as one would expect from the WAAS ionospheric model (see Table 1).

Given that the asymptotic properties of ionospheric modeling errors are better understood than those of multipath errors, it is instructive to eliminate, insofar as possible, all ionospheric modeling errors from the along-track error histories. To this end, a differential code phase GNSS technique was applied whereby the navigation solution was based on double-difference pseudorange measurements using data from a nearby reference station at a precisely known location. Such double differencing over a short 1-km baseline eliminates virtually all ionospheric and tropospheric errors, but does nothing to reduce vehicle-side multipath. Thus, one can empirically examine multipath effects in isolation from ionospheric effects.

**Figure 15:**
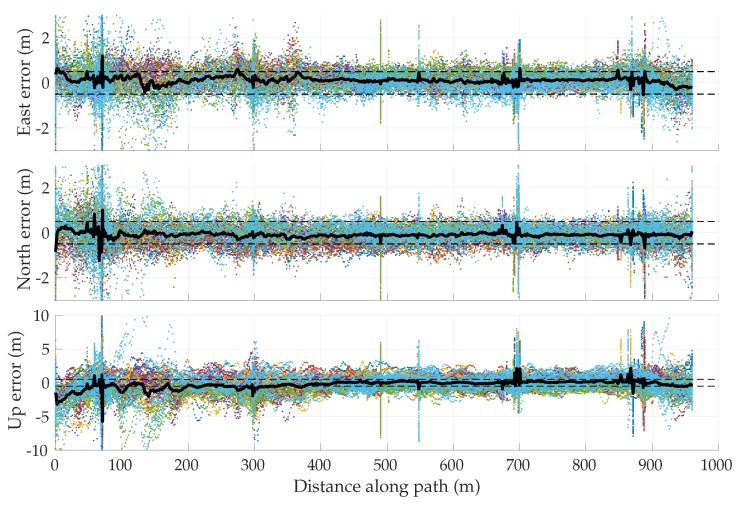
Errors in double-differenced pseudorange-based position estimates with respect to ground truth in the east, north, and up directions. Different colors distinguish data from six different campaigns. Dashed reference lines are drawn at ±50 cm. The solid black lines show the mean positioning error over the six campaigns. The error standard deviation in the east and north directions is ∼0.9 m over the first 200 m along the path and ∼0.4 m over the rest. In the up direction, the standard deviation is ∼1.9 m over the first 400 m and ∼1 m over the rest.

Figure 15 shows the results of this study based on all six data capture campaigns. Note that biases for all components are much smaller. It appears that for the test route chosen, non-zero-mean horizontal errors in the enhanced code phase positions were almost entirely driven by ionospheric modeling errors, and not by persistent effects of multipath due to the permanent structures along the test route. This is broadly consistent with the analyses presented earlier in this paper on position-domain biases due to ionospheric and multipath errors. However, it does appear that a bias due to multipath remained in the vertical direction over the first 400 m, even when ionospheric errors were removed. Apparently, the arrangement of buildings over this segment caused non-line-of-sight effects that did not average away. Mercifully, horizontal errors, which appear to be close to zero-mean over the six campaigns, matter most for high-accuracy digital mapping, since obstacle avoidance and vehicle coordination are largely 2-D problems, and since multiple vehicles can straightforwardly agree on a particular feature’s relative vertical position from an inferred road surface.

Based on Figure 15, one can conclude that multi-session averaging with a sufficiently accurate ionospheric model, such as the Fast PPP model, yields sub-50-cm global referencing accuracy for digital maps in the horizontal plane with code-phase-based GNSS, even in the presence of persistent multipath.

### 5.4. Multi-Session Mapping Results

**Figure 16:**
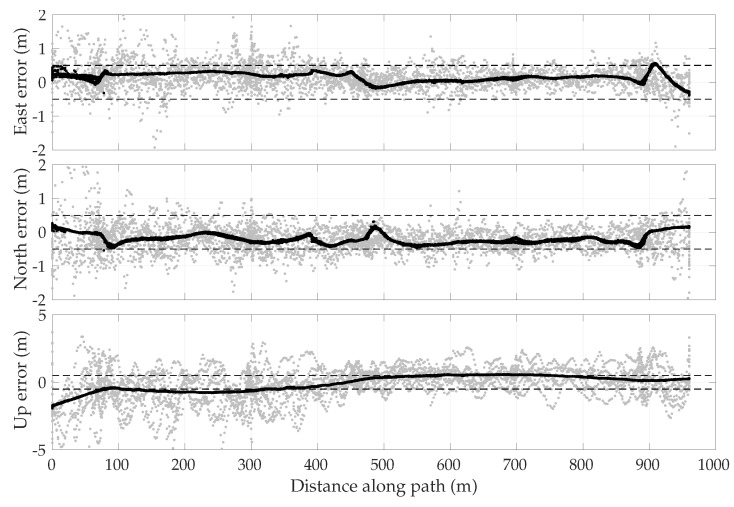
Errors in GEOSLAM’s estimate of the primary antenna position (in black) with respect to ground truth in the east, north, and up directions for eight mapping sessions from four different data collection campaigns. The errors in double-differenced pseudorange-based primary antenna position estimates for each of the eight sessions, fed as measurements to GEOSLAM, are plotted in gray for reference. Dashed reference lines are drawn at ±50 cm.

GEOSLAM processed two laps/sessions of data from each of the four campaigns conducted on 4 and 5 June, fusing the visual data from the captured images with the double-differenced pseudorange-based position estimates of the primary antenna. Figure 16 summarizes the result from GEOSLAM’s multi-session GNSS-aided-visual SLAM. The black data points denote the difference between the ground truth trajectory of the primary GNSS antenna and GEOSLAM’s estimate of the same in local east, north, and up directions for all eight sessions. The gray data points denote the difference between the ground truth trajectory of the primary GNSS antenna and the coincident double-differenced pseudorange-based estimate of the same for all eight sessions.

As one might expect, the error in GEOSLAM’s estimate of the antenna position was approximately the same as the average double-differenced pseudorange-based error over eight sessions. Furthermore, due to the approximately zero-mean nature of the double-differenced pseudorange-based estimates, the GEOSLAM esimate of the trajectory was within 50 cm of the truth trajectory in the horizontal plane. Note that the error in GEOSLAM’s position estimate was highly repeatable over eight different sessions, so much so that it appears there is a single black trace in Figure 16, while in truth eight independent traces were plotted. This indicates that (i) the localization of the vehicle within the visual map was highly precise: GEOSLAM made the same errors with respect to ground truth over eight different sessions; and (ii) the visual map was merged across eight sessions from four different campaigns: if the maps from any two campaigns were not merged through visual matching of features, then the GNSS position estimate for a keyframe from one campaign would not affect another keyframe from a different campaign since they would not be covisible, and thus the eight black traces in Figure 16 would not overlap.

## 6. Conclusions

The accuracy limits of collaborative global referencing of digital maps with standard GNSS were explored through simulation and real data. The asymptotic average of position errors due to thermal noise, satellite orbit and clock errors, and tropospheric modeling errors were assumed to be negligible. It has been shown that the position error due to inaccurate ionospheric modeling may lead to persistent dm-level biases in the horizontal position if the corrections are sourced from the IGS GIM, but other recent models such as the Fast PPP IONEX GIM perform better in this regard. Multipath errors persist with multiple mapping sessions through the same urban corridor and may not be zero mean. With adequate multipath exclusion, persistent multipath biases may be reduced below 50 cm on average. In conclusion, sub-50-cm accurate digital mapping has been shown to be feasible in the horizontal plane after multiple mapping sessions with code-phase-based GNSS, but larger biases persist in the vertical direction. A globally-referenced electro-optical SLAM pipeline, termed GEOSLAM, has been detailed and demonstrated to achieve sub-50-cm horizontal localization accuracy in a moderate urban environment by incorporating code-phase-based GNSS position estimates in the visual SLAM framework and jointly optimizing maps merged across time-separated sessions.

## Figures and Tables

**Table 1:** Long-term average position error due to ionospheric model errors (ϕ denotes station latitude). IGS: International Global Navigation Satellite System (GNSS) Service; PPP: precise point positioning; WAAS: Wide Area Augmentation System; CONUS: contiguous United States; IONEX: ionosphere-map exchange format.

Ionosphere Model	Region	East (m)	North (m)	Up (m)
IGS	$\phi \geq 30^{\circ }$	0.0107	−0.2129	0.6733
	$30^{\circ }>\phi >-30^{\circ }$	−0.0651	−0.0692	1.5467
	$\phi \leq -30^{\circ }$	0.0237	0.2450	0.3355
WAAS	CONUS	−0.0048	−0.2916	−0.1248
Fast PPP IONEX	$\phi \geq 30^{\circ }$	−0.0042	−0.0099	−0.0122
	$30^{\circ }>\phi >-30^{\circ }$	−0.0390	0.0013	−0.3053
	$\phi \leq -30^{\circ }$	−0.0325	−0.0087	0.0309

**Table 2:** Some urban scenario parameters.

Distance from road center to buildings	24 m	Distance from road center to vehicle	5 m
Mean distance between road center and trees	20 m	Antenna height	2 m
Mean building width	30 m	Building width standard deviation	25 m
Mean building height	40 m	Building height standard deviation	20 m
Probability of gap between buildings	0.5	Mean gap width	30 m
Mean distance between trees	60 m	Mean distance between poles	25 m

**Table 3:** 95-percentile horizontal errors.

Averaging Ensemble Size:		1	2	4	8	16	32	50	100
**Ideal**	0–60 s average (m)	1.5910	1.1262	0.7902	0.5488	0.4078	0.3090	0.2696	0.2147
	13–19 s average (m)	2.5925	1.7809	1.2136	0.8927	0.6416	0.4145	0.3544	0.2609
**NIS**	0–60 s average (m)	1.7851	1.2795	0.9245	0.6588	0.5169	0.4175	0.3920	0.3526
	13–19 s average (m)	3.1217	2.1953	1.5467	1.1720	0.8456	0.6470	0.5950	0.4702
